# Is Overlain Display a Right Choice for AR Navigation? A Qualitative Study of Head‐Mounted Augmented Reality Surgical Navigation on Accuracy for Large‐Scale Clinical Deployment

**DOI:** 10.1111/cns.70217

**Published:** 2025-01-16

**Authors:** Jian Ye, Qingwen Chen, Tao Zhong, Jian Liu, Han Gao

**Affiliations:** ^1^ Department of Neurosurgery, Affiliated Qingyuan Hospital Guangzhou Medical University, Qingyuan People's Hospital Qiangyuan China; ^2^ Department of Neurosurgery The First Affiliated Hospital of Guangdong Pharmaceutical University Guangzhou China

**Keywords:** accuracy, augmented reality, head‐mounted, human factor, overlain display, transformation, user‐dependent

## Abstract

**Background:**

During the course of the past two decades, head‐mounted augmented reality surgical navigation (HMARSN) systems have been increasingly employed in a variety of surgical specialties as a result of both advancements in augmented reality–related technologies and surgeons' desires to overcome some drawbacks inherent to conventional surgical navigation systems. In the present time, most experimental HMARSN systems adopt overlain display (OD) that overlay virtual models and planned routes of surgical tools on corresponding physical tissues, organs, lesions, and so forth, in a surgical field so as to provide surgeons with an intuitive and direct view to gain better hand–eye coordination as well as avoid attention shift and loss of sight (LOS), among other benefits during procedures. Yet, its system accuracy, which is the most crucial performance indicator of any surgical navigation system, is difficult to ascertain because it is highly subjective and user‐dependent. Therefore, the aim of this study was to review presently available experimental OD HMARSN systems qualitatively, explore how their system accuracy is affected by overlain display, and find out if such systems are suited to large‐scale clinical deployment.

**Method:**

We searched PubMed and ScienceDirect with the following terms: head mounted augmented reality surgical navigation, and 445 records were returned in total. After screening and eligibility assessment, 60 papers were finally analyzed. Specifically, we focused on how their accuracies were defined and measured, as well as whether such accuracies are stable in clinical practice and competitive with corresponding commercially available systems.

**Results and Conclusions:**

The primary findings are that the system accuracy of OD HMARSN systems is seriously affected by a transformation between the spaces of the user's eyes and the surgical field, because measurement of the transformation is heavily individualized and user‐dependent. Additionally, the transformation itself is potentially subject to changes during surgical procedures, and hence unstable. Therefore, OD HMARSN systems are not suitable for large‐scale clinical deployment.

## Graphical abstract

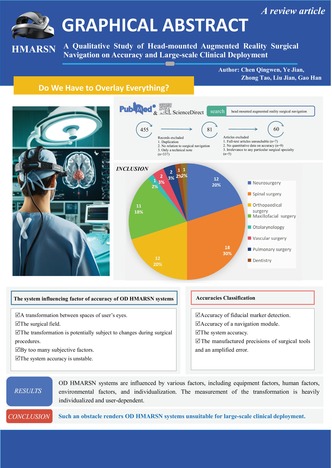

## 1 Introduction

Augmented reality (AR) is an interactive experience that combines the real world and computer‐generated content. In surgical practices, augmented reality surgical navigation (ARSN) generally refers to the superimposition and display of certain information over the surgical field prior to and/or during surgeries to guide surgeons' operations. Certain information can be a patient's two‐/three‐dimensional virtual models reconstructed from the patient's medical images (segmented and/or fused if necessary) as well as planned and real‐time routes of surgical tools, while the surgical field can be either acquired by cameras (video see‐through, or VST) installed on a head‐mounted device (HMD) or viewed directly by surgeons (optical see‐through, or OST) through projector screens of an HMD. Depending on whether virtual models are aligned with their physical counterparts in the surgical field, HMARSN can be classified into OD and adjacent display (AD) as well.

ARSN is not new. It can be traced back to 1968, when Ivan Sutherland demonstrated an OST HMD using miniaturized cathode‐ray tubes as stereoscopic displays and either an ultrasonic or a mechanical tracker [1], and another ARSN system was also designed to display computed tomography (CT) imaging in an operating room microscope in the 1980s [2]. Apart from HMDs, the other form of ARSN is casting images of virtual models onto patients using a standalone projector. It was not until the emergence of commercial HMDs such as Google Glass, Epson's AR glasses of BT‐series, Magic Leap, and Microsoft Hololens that experimental HMARSN has been increasingly built up and tested by medical practitioners in a wide variety of surgical specialties, in which ODs are widely utilized. Furthermore, several HMARSN systems have become commercially available, for example, xVision from Augmedics, Knee++ from Pixee Medical, as well as Caduceus S from Main Orthopaedic Biotechnology; yet none of them adopts ODs for either preoperative registration or intraoperative navigation. It is therefore the aim of this study to analyze how ODs affect the accuracies of HMARSN systems, and whether OD HMARSN systems can be applied in large‐scale clinical deployment.

## 2 Methodology

### 2.1 Search Strategy and Study Selection

We conducted our search across the PubMed and ScienceDirect databases using the search terms: “head mounted augmented reality surgical navigation.”

### 2.2 Eligibility Criteria and Selection Process

**FIGURE 1:**
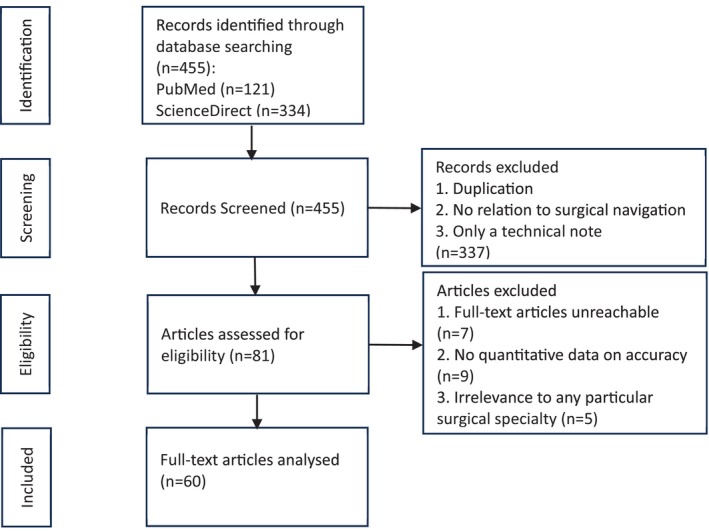
Selection process.

By the end of November 2023, the search yielded 445 records, of which 81 papers remained after screening that removed duplications, irrelevant papers, and non‐article records. We further checked their eligibility and removed 20 papers due to unobtainable full texts (7), lack of quantitative accuracy data (9), and irrelevance to any particular surgical specialty (*n* = 5). Finally, 60 papers were included in this study. The selection process is illustrated in Figure 1.

## 3 Distribution of Specialties

**FIGURE 2:**
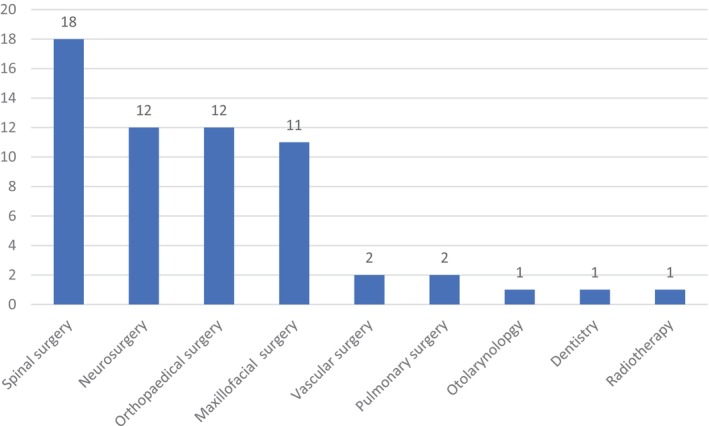
Distributions of analyzed papers in the study by surgical specialty.

Across the 60 papers we analyzed, most HMARSN systems were applied in surgical specialties handling rigid bodies (56). The four largest ones were spinal surgery (18), orthopedic surgery (12), neurosurgery (12), and maxillofacial surgery (11). The rest included otolaryngological surgery (1), pulmonary surgery (2), vascular surgery (2), dentistry (1), and radiotherapy (1). Distributions of all papers as per surgical specialties are depicted in Figure 2.

Papers that are found by different specialties are listed in Table 1.

**TABLE 1:** HMARSN in different surgeries.

Author	Year	HMD	OD	Amount of data	Subject type	Navigational accuracy	Accuracy type
*Spinal surgery* All procedures in this subgroup are pedicle screw insertions except one for sacral‐alar‐iliac (SAI) screws for lumbo‐pelvic fixation [3] TRE: Target Registration Error PVE: Planned vs. executed trajectories GRS: Gertzbein–Robbins grading Scale MAPW: The minimal distance of the drill axis to the pedicle wall. It is defined as the shortest distance between the drill axis and the pedicle wall that could be measured along the entire trajectory. Larger values correspond to a more centric drill, which allows to use a bigger screw diameter without perforation of the pedicle cortex ROS: Rate of success without perforation							
Jacob T. Gibby et al. [4]	2018	Hololens	Yes	36 needles	Phantom	2.50 mm	TRE
Liebmann Florentin et al. [5]	2019	Hololens	Yes	10 screws on 5 vertebrae	Phantom	2.77 ± 1.46 mm, 3.38° ± 1.73°	PVE
Fabio Müller et al. [6]	2019	Hololens	Yes	3 lumbar cadaver spines	Phantom	3.4 ± 1.6 mm, 4.3° ± 2.3°	PVE
Camilo A. Molina et al. [7]	2020	xVision	Both	6 screws on 1 patient	Patient	2.07 (95% CI: 1.62–2.52 mm), 2.41 (95% CI: 1.57°–3.25°).	PVE
Vivek P. Buch et al. [8]	2020	Hololens	Yes	7 patients	Patient	2.30 ± 0.58 mm	TRE
Cyrill Dennler et al. [9]	2020	Hololens	Yes	40 screws in 8 lumbar vertebrae	Phantom	4.2 ± 1.8 mm for novice5.0 ± 1.4 mm for expert	MAPW
Daniel S Yanni et al. [10]	2021	Magic Leap	No	192 screws	Phantom	98.4%	GRS
Ann Liu et al. [11]	2021	xVision	Both	205 screws	Patient	98%	GRS
Mazda Farshad et al. [12]	2021	Hololens	Yes	4 screws	Patient	3.5 ± 1.9 mm, 7.3° ± 3.6°	PVE
Cyrill Dennler et al. [3]	2021	Hololens	Yes	40 holes on 1 pelvic model	Phantom	97.5%	ROS
Henrik Frisk et al. [13]	2022	Magic Leap	Yes	48 screws	Phantom	1.9 ± 0.7 mm (entry), 1.4 ± 0.8 mm (tip), 3.0° ± 1.4°	PVE
Chih‐Chang Chang et al. [14]	2022	Caduceus S	Both	24 screws on 1 cadaver	Cadaver	87.5%	GRS
Alejandro Martin‐Gomez et al. [15]	2023	Hololens	Yes	168 screws	Phantom	2.79 ± 1.52 mm, 4.56° ± 2.49°	PVE
Eun Kyung Jun et al. [16]	2023	Hololens	Yes	10 screws	Phantom	1.7 ± 2.3 mm	TRE
Florentin Liebmann et al. [17]	2023	Hololens	Yes	10 screws	Phantom	~1–2 mm	Median TRE
Bing Cao et al. [18]	2023	Hololens	Yes	83 screws in 5 cadavers	Cadaver	98.80% based on CT and 72.29% based on C‐ARM ≤ 2 mm	GRS
José Miguel Spirig et al. [19]	2020	Hololens	Yes	8 k‐wires	Cadaver	5.99 ± 3.60 mm	PVE
Huixiang Wang et al. [20]	2016	nVisor ST60	Yes	12 screws	Cadaver	2.7 ± 1.2 mm at entry point, 3.7 ± 1.1 mm at tip, 2.9° ± 1.1°	PVE
*Neurosurgery* Procedures in this subgroup include punctual‐styled operations as well as craniotomies *A calibrated tape is attached on grooves of a phantom, and Euclidean distances of operations are compared to them. It is equivalent to TRE TVE: Target Visualization Error FRE: Fiducial Registration Error							
Qichang Sun et al. [21]	2020	Hololens	Yes	Phantom, 8 cases, 62 titanium fiducials	Phantom	1.30 ± 0.39 mm	TRE
Francis X Creighton, John Carey et al. [22]	2020	Hololens	Yes	10 points on a bone phantom	Phantom	10.62 ± 5.90 mm	TRE.
Sara Condino et al. [23]	2021	VOSTARS	Yes	520 measurements	Phantom	1.30 ± 0.6 mm	TVE
T Fick et al. [24]	2021	Hololens	Yes	6 registration errors on 3 patients	Patient	8.5 mm	FRE
Marcin Majak et al. [25]	2021	Hololens	Yes	24 trials	Phantom	2.50 ± 0.93 mm	FRE
Y Gao et al. [26]	2022	Hololens	Yes	Phantom	Phantom	1.036 ± 0.081 mm	PVE
Theo Demerath et al. [27]	2022	Magic Leap	Yes	71 patients	Patient	Euclidean median 3 mm	PVE
Wendell Gibby et al. [28]	2022	Hololens	Yes	15 procedures	Phantom	2.3° ± 1.28°, 3.62 ± 1.71 mm	PVE
Ziyu Qi et al. [29]	2023	Hololens	Yes	6 points on a phantom	Phantom	3.7 ± 1.7 mm	TRE
Ye Li et al. [30]	2023	Hololens	Yes	14 out of 30 patients	Patient	5.46 ± 2.22 mm	PVE
Zeyang Zhou et al. [31]	2022	Hololens	Yes	10 patients	Patients	1.65 mm	PVE
Federica Ruggiero et al. [32]	2023	Hololens	Yes	6 cuts on nasal and frontal osteotomies, respectively	Phantom	~1.5 mm	TRE*
*Orthopedic surgery* Procedures in this subgroup include various operations on bones, such as ostectomy [33, 34, 35], tumoral intervention [36], PFA [37] among others ǂ It is calculated by comparing the poses of the endoscope after performing the positioning task to the respective ground‐truth endoscope poses, which is equivalent to PVE ◊ It is calculated by deviation (mm) in the normal direction from estimated cut plane per subject trial—overall roughness * It is compatible with methodology of ASTMF2554 [38] ** It is a localization error, which is similar to TRE. Albeit it is practiced on a patient, accuracy of PVE is not provided							
Joerg Traub [39]	2006	Sys. Designed by Sauer et al. [40] /w a 3rd camera	Yes	112 measurements	Phantom	1.5–3.1 mm	PVE
Xue Hu et al. [41]	2015	Hololens /w visible‐light binocular camera	Yes	10 users	Phantom	4.90 ± 1.04 mm, 5.96° ± 2.22° for VST, and 4.36 ± 0.80 mm 5.65° ± 1.42° for OST	PVE
Rafael Moreta‐Martinez et al. [36]	2018	Hololens	Yes	15 points	Phantom	~3 mm	TRE**
Yuan Gao et al. [33]	2019	Hololens	Yes	9 points	Phantom	3.26 ± 1.40 mm	TRE
Pascal Kiarostami et al. [34]	2020	Hololens	Yes	4 points	Phantom	~2 mm	PVE
M. Koyachi et al. [35]	2020	Hololens	Yes	26 points	Patient	Median 0.56 mm	TRE
Hisham Iqbal et al. [37]	2021	Hololens	Yes	10 phantoms	Phantom	1.06 mm	PVE◊
Andrea Teatini et al. [42]	2021	Hololens	Yes	A validation phantom & a patient phantom	Phantom	8.22 ± 2.27 mm for the 1st; and 10.89 ± 3.78 mm for the 2nd	PVE*
Puxun Tu et al. [43]	2021	Hololens	Yes	19 trials	Phantom	1.61 ± 0.44 mm, 1.46° ± 0.46°	TRE
Tianyu Song et al. [44]	2022	Hololens	No	5 alignment tasks	Phantom	6.6 ± 3.3 cm, 51.3° ± 45.4°	PVE ǂ
J Tomás Rojas [45]	2022	NG	No	NG	Patient	NG	NG
Philipp Kriechling et al. [46]	2023	Hololens	Yes	12 shoulders	Patient	3.5 ± 1.7 mm from entry points, 3.8° ± 1.7°	PVE
*Maxillofacial surgery* Oral‐maxillofacial, craniomaxillofacial procedures are included in this subgroup ⌂ It is computed as deviations of vertical differences between actual osteotomy and preoperative designed osteotomy planes, which is equivalent to PVE * It is computed by linear distances between the expected and real positions on the phantom, which is equivalent to PVE ** It is computed by absolute differences in poses (position and orientation) between tracked and planned cubes, which is equivalent to PVE ǂ Euclidian distance between the final position of instrument's tip and the target landmark. The number is average of fixed and floating landmarks. It is in fact a test of accuracy of a navigational module ◊ It is computed as the mean difference of planned and executed planes, which is equivalent to PVE ○ It is computed by superimposed discrepancy between postoperative 3D craniomaxillofacial model and preoperative virtual plan, which is equivalent to PVE							
Miao Qu et al. [47]	2014	NG	Yes	2 patients	Patient	1.22 ± 0.15 mm	PVE⌂
Piotr Pietruski et al. [48]	2019	BT‐200	Yes	126 cases on 21 phantoms	Phantom	1.65 ± 0.88 mm, 4.94° ± 4.62°	PVE⌂
Shaofeng Liu et al. [49]	2023	Hololens	Yes	9 group tests	Phantom and animal	1.62 ± 0.38 mm, 3.68° ± 0.71°	PVE⌂
Christina Gsaxner et al. [50]	2020	Hololens	Yes	11 experts on a phantom	Phantom	74.8 ± 15.9	Usability scale score
Claudia Scherl et al. [51]	2021	Hololens	Yes	1 patient	Patient	1.3 cm	TRE
Giovanni Badiali et al. [52]	2014	WARM	Yes	7 points	Phantom	1.70 ± 0.51 mm	PVE*
Jene W. Meulstee et al. [53]	2019	Hololens	Yes	5 cubes	Measuring board	2.3 mm	PVE**
Laura Cercenelli et al. [54]	2020	VOSTARS	Yes	4 trajectories	Phantom	~ ± 1.0 m	Traced trajectory curves falling within the inspection window
H. H. Glas et al. [55]	2021	Hololens	Yes	3 fixed landmarks 6 floating landmarks 3 trajectories	Phantom	~1.88 mm	Acc. of nav. moduleǂ
Zu‐Nan Tang [56]	2022	Hololens	No	7 patients	Patient	1.68 ± 0.92 mm	PVE◊
Liu, K. et al. [57]	2021	Hololens	No	Hundreds of points on 5 patients	Patient	1.442 ± 0.234 mm	PVE○
*Vascular surgery* Two procedures are included for vascular surgery.							
Taoran Jiang et al. [58]	2020	Hololens	Yes	5 points /w various angles & ambient light	Phantom	~2–3 mm	TRE
Mitchell Doughty et al. [59]	2022	Hololens	Yes	10 patients	Patient	0.98 mm	TRE
*Pulmonary surgery* Two procedures are included for pulmonary surgery. * It is computed by comparing positions of implanted markers and those of actual lesions, which is equivalent to PVE							
Chengqiang Li et al. [60]	2021	Hololens	No	4 canine models	Phantom	1.9 ± 1.7 mm	PVE*
Chengqiang Li et al. [61]	2023	Hololens	Yes	10 nodules in 10 patients	Patient	Median: 5.8 mm	PVE

Besides papers in the aforementioned specialties, one paper is found in each of the following specialties.

### 3.1 Dentistry

In [62]. Hololens was employed and OD was adopted. PVEs were calculated from 242 dental implantations on a phantom, and gained 1.31 ± 0.67 mm and 1.36 ± 0.67 mm in entry and tip linear accuracies, respectively, with an angular accuracy of 3.72° ± 2.13° [62].

### 3.2 Otolaryngological Surgery

In [63], excisions of two median neck cysts and one branchial cyst were performed on three patients assisted by OD. Only TREs were recorded.

### 3.3 Radiotherapy

Utilizing Hololens by employing OD [64] focuses on aligning and correcting the patient's posture and achieved a TRE of 3.0 ± 1.5 mm when tested on a mannequin.

## 4 Analysis of System Accuracy of OD HMARSN for Large‐Scale Clinical Deployment

### 4.1 A Brief Description of the Surgical Navigation Process

Generally speaking, surgical navigation systems do not differ from their counterparts in other applications in terms of both identifying geometric relationships among multiple coordinate systems (spaces), and hence performing a series of transformations accordingly in a real time. As far as an AD HMARSN system is concerned, the following steps are executed.

**FIGURE 3:**
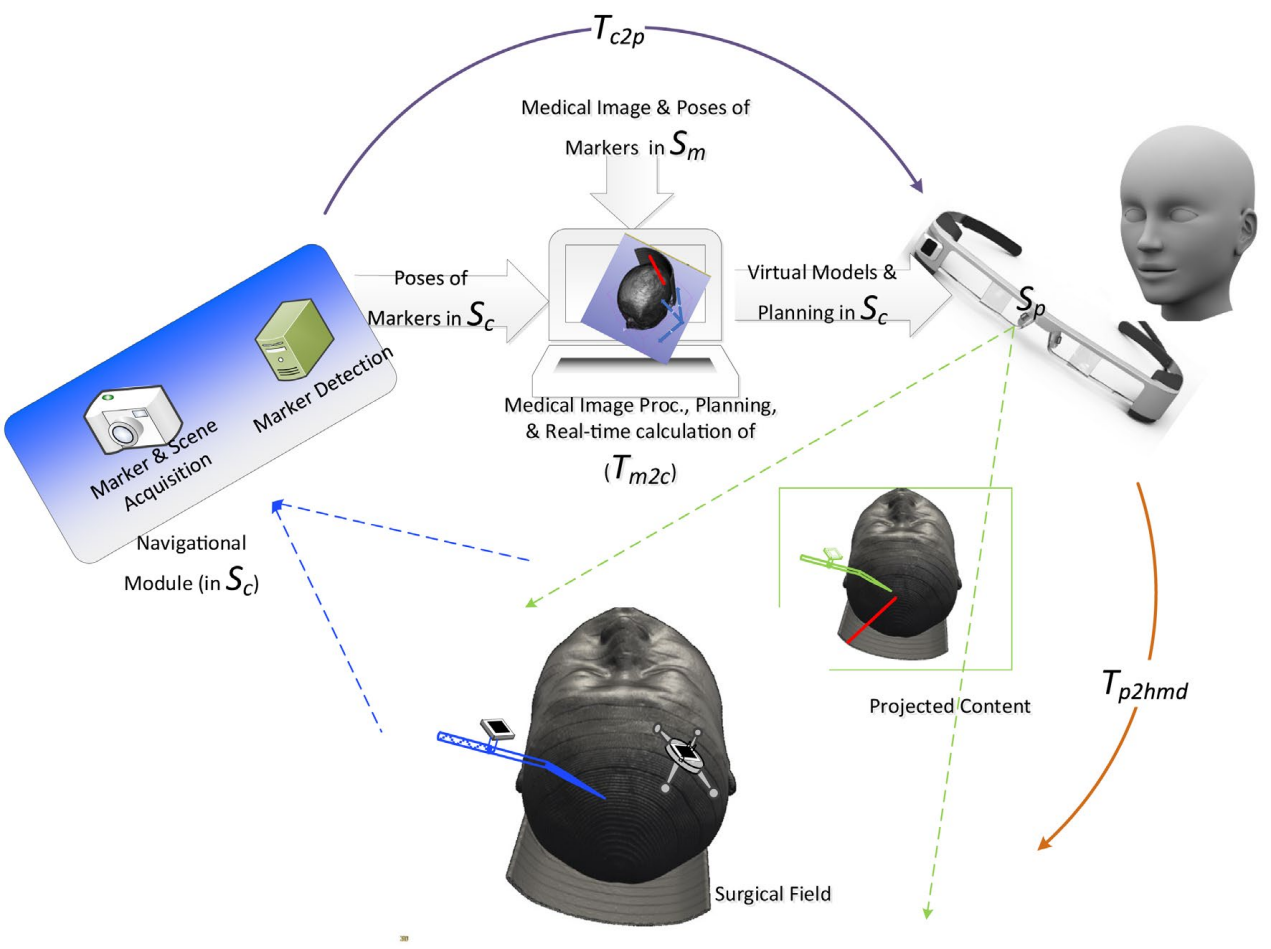
The entire navigation process. Red line indicates a planned route; blue and green dashed lines show acquisition of the surgical field and field of projected virtual models, respectively; purple curve shows the transformation between Sc and Sp, that is, $T_{c2p}$, while orange curve shows the calibrated transformation $T_{p2\mathrm{hmd}}$ between Sp and the surgical field.

1. During preoperative registration, poses of one set of markers are defined in Sm space of a patient's medical image;
2. During navigation, the following steps are executed in real time:
  2.1 Poses of another set of corresponding markers attached to the patient in Sc space are detected;
  2.2 A transformation $T_{m2c}$ is calculated based on aforementioned two sets of markers;
  2.3 Virtual models generated from medical images and planned routes of surgical tools in medical images are mapped into Sc by $T_{m2c}$, and then cast through projector screens of an HMD together in front of a surgeon's eyes;
  2.4 Poses of surgical tools are also derived by detecting markers attached to them, and then cast through projector screens simultaneously. Doing so enables the surgeon to make them follow their planned counterparts so as to accomplish intraoperative tracking;
  2.5 Optionally, a $T_{c2p}$, which is the fixed geometric relationship between Sc space and Sp, that is, space of the projector screens, may also be incorporated in order to map the transformed virtual models residing in Sc further into Sp by $T_{c2p}*T_{m2c}$. Such a mapping is utilized for correcting delicate differences of surgeon's view point and the view point of image acquisition;

The entire process is depicted in Figure 3. Note that determinations of all aforementioned transformations are objective and minimally user independent, because markers in both Sc and Sm, and consequently $T_{m2c}$, are detected and computed by algorithms; while $T_{c2p}$ has been fixed when the HMD is manufactured. It can therefore be asserted that any given AD HMARSN system holds a consistent and objective system accuracy.

Yet, an additional transformation $T_{p2\mathrm{hmd}}$ must be considered and measured for an OD HMARSN system. $T_{p2\mathrm{hmd}}$ tends to overlay the content of the projector screen of the HMD to their physical counterparts in the surgical field. Consequently, the surgeon can move the surgical tools along their virtual planned routes accordingly. The OD configuration was adopted in 55 of the 60 papers involved in the present study, and all such systems are experimental ones.

### 4.2 Levels of Accuracies

Prior to analyzing if the $T_{p2\mathrm{hmd}}$ violates the objectivity of accuracy measurement, classifications of accuracies must be outlined. The system accuracy of a typical surgical navigation system, whether it is a traditional one (including surgical robot) or an AR HMARSN, can be broken down into several levels from two perspectives.

From an engineering perspective, three levels of accuracy should be delineated: accuracy of fiducial marker detection (if a system adopts markers), accuracy of a navigation module, and the system accuracy. The accuracy of fiducial marker detection refers to the accuracy of detected poses (position and angle) of one or a set of fiducial markers. It is the most fundamental accuracy, since the poses of both registered models and tracked surgical tools are derived from the poses of markers. Popular choices of fiducial markers include visible black and white planar or cubic markers, colored spherical markers, active or passive infrared markers, and electromagnetic markers. Hybrid fiducial marker detection is also feasible, which was utilized in [44]. Different types of fiducial markers utilized across the analyzed papers are summarized in Table 2, in which visible black and white markers and infrared markers are adopted dominantly. The accuracy of fiducial marker detection primarily relies on algorithmic design, the marker's manufacturing precision as well as the hardware configurations of image acquisition systems such as infrared or visible‐light cameras. Marker‐free scenarios are discussed later in the preoperative registration.

**TABLE 2:** Summary of types of markers applied in the analyzed papers.

Type of marker	Number of application
Visible black and white markers (QR Code)	21
Infrared markers	26
Colored spherical markers	3
Electromagnetic markers	2
Hybrid markers	2
No marker used	6

The accuracy of the navigation module includes not only that of fiducial marker detection, but also that of the manufactured precision of surgical tools and installation of markers as well as amplified error. For example, the tip position of a straight surgical tool (a probe, a k‐wire, a biopsy needle, etc.) that touches the patient is often inferred from a fixed position where markers are attached. Broadly speaking, the more distant the two positions are, the more the error of fiducial marker detection will be amplified; hence, the accuracy of the navigation module is usually lower than that of fiducial marker detection.

As for the system accuracy, a further decrease is caused primarily owing to both reprojection errors of calculated $T_{m2c}$ during the preoperative registration and intraoperative tracking as well as inherent errors in mechanical systems if robotic arms are adopted.

It can be readily concluded that all the three levels of accuracies are objective and minimally user‐dependent, that is, they are merely dependent on algorithmic design and the manufacturing precision of devices and tools. Accuracies and sources of errors are summarized in Table 3.

**TABLE 3:** Types of accuracy and sources of error.

Type of accuracy/Error source	Fiducial marker detection	Navigational module	Navigational system
Marker detection	Yes	No	No
Tool's manufacturing	Yes	Yes	No
Positional amplification	Yes	Yes	No
Preoperative registration	Yes	Yes	Yes
Tracking reprojection	Yes	Yes	Yes
Mechanical system	Yes	Yes	Yes

From a clinical perspective, on the other hand, two levels of accuracies should be taken into account: accuracies of preoperative registration and of intraoperative tracking. The system accuracy is aggregated from both. Two methods are usually adopted in preoperative registration, namely, the point‐pair–based method and the surface matching method. The former requires a user to select multiple pairs of points (usually positions of fiducial markers) on both the patient and her/his reconstructed medical image, while the latter asks the user to scan the patient's surface by (1) a probe with attached markers, (2) a pen emitting class‐I medical laser as described in [65], or (3) structure light, so that a point cloud will be collected and matched with the patient's reconstructed medical image algorithmically. All the three methods are found in the analyzed papers, and they primarily depend on algorithmic design and manufacturing precision of devices. What is alternatively utilised for pre‐operative registration is manually aligning virtual models with physical scenes that were found in 3 papers, yet they have fallen out of the scope of our study due to lack of any gaugeable accuracies. They are mentioned here simply for completeness.

During the intraoperative tracking, various types of fiducial markers mentioned above are deployed, and algorithmic design of real‐time fiducial marker detection and of calculating $T_{m2c}$ dominate the tracking accuracy. It can therefore be concluded that both accuracies of the preoperative registration and of the intraoperative tracking are objective and minimally user‐dependent.

In summary, however accuracies are categorized, the system accuracy of typical surgical navigation systems is objective and minimally user‐dependent. In practice, it is usually measured by comparing planned routes of surgical tools in preoperative images and final positions of surgical tools in postoperative images of the same phantom or utilizing a third‐party high‐end measuring device. For instance, a 4K stereo camera system mounted on a high‐precision 5‐axis mechanical platform can be deployed to acquire images of final positions of a surgical tool and calculate its poses by computer vision. Since the relative pose of the platform, hence the camera, to the phantom is known, those poses can be translated into that of the phantom, and subsequently compared with their planned counterparts. Both methods for measuring the system accuracy are objective and minimally user‐dependent. A typical example is that in FDA's Summary for Caduceus S [66], in which how the first approach is adopted is clearly stated.

### 4.3 Accuracy of OD HMARSN

**FIGURE 4:**
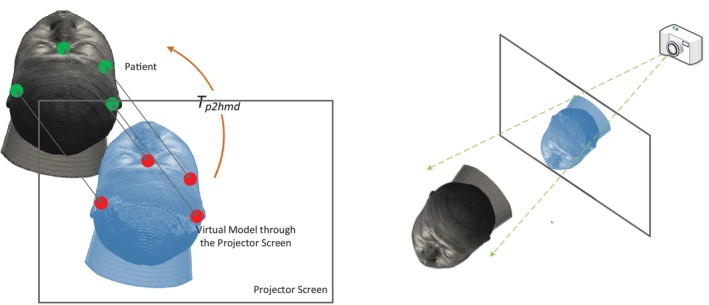
Left: The schematic diagram of basic calibration for an OD configuration. Right: The equivalent camera system.

The aforementioned analysis is valid for both traditional surgical navigation and AD HMARSN systems. As long as an OD HMARSN system is concerned, a calibration that derives $T_{p2\mathrm{hmd}}$ must be incorporated into the preoperative registration. A manual calibration of $T_{p2\mathrm{hmd}}$ is conducted by user's selection of multiple virtual points and their corresponding physical points in front of her/his eyes in turn, as stated in [21], when the user wears an HMD. The scheme is depicted in Figure 4(left). A single‐point active alignment method (SPAAM) and its variations for the calibration are presented with further improvements [40]. Furthermore, semi‐automatic [67] and automatic [68] calibrations are also available. The essence of all calibration methods is equivalent to locating a camera behind each projector screen of the HMD as depicted in Figure 4(right). The camera is intended to emulate user's eye. By adjusting the camera's parameters (intrinsic matrix $M_{\text{in}}$) and its relative pose (extrinsic matrix ${\left\lfloor R\;T\right\rfloor }_{\mathrm{ex}}$) to the projector screen, a series of images is acquired and examined so as to find a combination of $M_{\text{in}}*{\left\lfloor \begin{array}{cc} R & T \end{array}\right\rfloor }_{\mathrm{ex}}$ that has a projected virtual model overlain upon the acquired physical scene in front the projector screen. The combination is then regarded as the pose where the user shall wear the HMD, that is, $T_{p2\mathrm{hmd}}$. However, the calibration is heavily individualized and user‐dependent, because users vary in their eyes physiologically; the intrinsic matrix $M_{\text{in}}$, which represents the user's eyes, may alter, for example, when her/his ciliary muscle contracts or relaxes, under different circumstances; and the relative pose described by the extrinsic matrix ${\left\lfloor \begin{array}{cc} R & T \end{array}\right\rfloor }_{\mathrm{ex}}$ is not constant.

In addition, as $T_{p2\mathrm{hmd}}$ are incorporated into the intraoperative tracking, a variety of optical, mechanical, and human factors contributes to its changes during the course as well. It follows that $T_{p2\mathrm{hmd}}$ during the navigation may not be the one calibrated a priori, that is, is unstable. Multiple papers in our study reported those factors including small field of view [22, 32, 54], limited/incompatible focal range of hologram [42, 50], stability and drift of hologram [5, 8, 9, 24], potential movement of HMD in reference to user's head (or equivalently, patient's movement) [12, 25], dizziness and fatigue [30, 43, 57], system reliability [12, 48], perceptual conflicts and inattentional blindness [37], and user‐dependent depth perception [48, 50, 56]. In particular, perceptual limits of HMD were discussed aided with an experiment in [69], and conclusions were made that even if there was a growing interest in using commercial OST HMD, for guiding high‐precision manual tasks, attention should be paid to the limitations of the available technology not designed for the peripersonal space.

Human factors, in particular, were emphasized extensively in [70], in which the following 13 factors are most related to preoperative attainment of $T_{p2\mathrm{hmd}}$ and its potential changes in the intraoperative tracking as follows:

- Individually different visual processing capabilities between dominant and non‐dominant eyes;
- Perceived comfort level when wearing optical‐see‐through‐HMD;
- Perception of spatial relationships between real and virtual objects;
- Personal degree of perceived immersion;
- Error‐prone and cognitively demanding mental mapping of 2D image data to 3D word;
- Distraction;
- Impaired intraoperative navigation abilities due to absence of visual aids;
- Limited mental information processing abilities;
- Loss of concentration;
- Visual fatigue;
- Missing/impaired depth perception;
- Unfamiliar/cognitively demanding hand–eye coordination;
- Error‐prone manual tool adjustment;

Consequently, measuring $T_{p2\mathrm{hmd}}$ to facilitate the use of OD HMARSN systems is a heavily individualized and user‐dependent process. Furthermore, $T_{p2\mathrm{hmd}}$ is subject to changes during the intraoperative tracking; hence, the system accuracy of OD HMARSN systems becomes highly unstable, and a minimal user dependence cannot be realized.

The conclusion above can be further verified by the reported accuracies in the analyzed papers, in which the OD is applied most prevalently (53/60). Considering the numbers of trials (ranging from hundreds to merely several), the ways in which their accuracies were measured, and the subject types (varying in phantoms, patients, various parts of cadavers) presented in those papers are quite diverse, and no regulatory body participated, it is impossible to conduct any quantitative statistical analysis Nonetheless, we adopt PVEs (utilized in 25 papers) to estimate their overall system accuracy roughly, because PVE is most similar to the method used by the FDA as stated above [66].

Average PVE across the largest four specialties are 3.32 mm for spinal surgery, 2.69 mm for neurosurgery, 3.39 mm for orthopedic, and 1.67 mm for maxillofacial, respectively; while a brute‐force mean PVE of 3.02 mm is given across all nine specialties. It is worth pointing out that only one paper [42] adopted a method for measuring the accuracy of navigational module of its OD HMARSN system similar to ATSM F2554 standard [66], and merely resulted 8.22 ± 2.27 mm on a validation phantom and 10.89 ± 3.78 mm on a patient phantom, respectively, which are far inferior to the accuracy of the state‐of‐the‐art navigational module, NDI's Polaris, which is around 0.5 mm.

If comparing them with commercially available systems by surgical specialties, the system accuracy of StealthStation S8 from Medtronic [71], which is designed for both neurosurgery and spinal surgery and often deemed a yardstick among commercial surgical navigation systems, is 1.45 ± 0.67 mm. The other two spinal navigation systems, xVision [72] and Caduceus S [66], achieved 1.98 ± 0.90 mm and 1.91 mm in system accuracies, respectively. In addition, most surgeons consider a less than 2 mm perforation as safe zone in pedicle screw insertion [73]. Furthermore, the paper [42] also observed statistically significant accuracies achieved by different users, which is a solid proof for serious effects of human factors on $T_{p2\mathrm{hmd}}$, and eventually, on the system accuracy.

The comparison offers a convincingly qualitative statement on comparative goodness of accuracies of analyzed OD HMARSN systems. It is therefore concluded that use of OD seriously affects the subjectiveness and user independence of the system accuracy, in particular by the unstable $T_{p2\mathrm{hmd}}$, and an OD HMARSN system is unsuited for large‐scale clinical deployment.

## 5 Conclusions

HMARSN systems have been increasingly experimented with in various surgical specialties, especially those handling rigid bodies, and most of them adopt OD. The study concludes that OD HMARSN systems are unsuitable for large‐scale clinical deployment due to their unstable system accuracy, rooted in using OD, which brings a heavily individualized and user‐dependent calibrated transformation between projector screen (user's eyes) and surgical field. In addition, the transformation is potentially subject to changes in the intraoperative tracking primarily caused by a variety of human factors. It should be recognized that the current analysis is mainly confined to accuracy‐related factors; yet, many other factors are closely related to large‐scale clinical deployment of HMARSN systems as well, such as weight, cost, sterilization, ease of training, comfort of use, EMC, and safety, to name a few. AR technology is no doubt on a fast‐paced developmental track, and provides a promising future for surgical navigation. It is therefore desirable to follow its improvements in the long term and conduct further studies so as to appreciate its potential.

## Author Contributions

**Qingwen Chen:** data and materials curation, select papers, writing – original draft. **Tao Zhong:** writing – original draft. **Jian Liu:** writing – review and editing. **Han Gao:** methodology, writing – original draft. **Jian Ye:** project administration, supervision, writing – review and editing.

## Conflicts of Interest

The authors declare no conflicts of interest.

## Data Availability

We searched PubMed and ScienceDirect with the following terms: head mounted augmented reality surgical navigation, and 445 records were returned in total. After screening and eligibility assessment, 60 papers were finally analyzed.
